# Supplementary material for: Attrition and associated factors among patients on chronic antihypertensive therapy at Mulago hospital, Uganda: A mixed method study
Source: PLoS One. 2026 Feb 26;21(2):e0327933. doi: 10.1371/journal.pone.0327933 (PMC12944796; doi:10.1371/journal.pone.0327933)
Supplement: S1 Appendix — (PDF) [file pone.0327933.s001.pdf]

S1 Appendix: Detailed methodological formulas

Survival data formulas for calculating sample size for factors associated with time to attrition

$$\Delta = \frac{\lambda_1}{\lambda_2} = \frac{\log \pi_1}{\log \pi_2}$$

$$e_2 = \frac{(Z_{\alpha/2} + Z_{1-\beta})^2}{\vartheta}\left[\frac{1+\vartheta\Delta}{1-\Delta}\right]^2$$

$$e_1 = \vartheta e_2$$

$$E = e_1 + e_2 = e_2(1+\vartheta)$$

Where,

π1 = Proportion surviving (retained on treatment) in those aged 41-52 (67.9%)

π2 = Proportion surviving (retained on treatment) in those 53-65 (79.6%)

Zα/2= Standard normal value corresponding to a 5% level of significance (1.96)

Z1-β = Standard normal value corresponding to 80% power of study (0.84) ϑ = Ratio of those aged 53-65 to those aged 41-52 (54/53)

e1= Expected events in those aged 41-52

e2=Expected events in those aged 53-65

E= Number of events

Substituting in the above formulae, 238 events were calculated, and subsequently these numbers of events were substituted in the formula below

$$N_{total} = \frac{(1+\vartheta)E}{(1-\pi_2)+\vartheta(1-\pi_1)}$$

Therefore, the required number of patient files was 905, and after accounting for 10% missing data, the minimum sample size for the second objective was 1006 patient files.
