# Supplementary material for: Attrition and associated factors among patients on chronic antihypertensive therapy at Mulago hospital, Uganda: A mixed method study
Source: PLoS One. 2026 Feb 26;21(2):e0327933. doi: 10.1371/journal.pone.0327933 (PMC12944796; doi:10.1371/journal.pone.0327933)
Supplement: S2 Appendix — (PDF) [file pone.0327933.s002.pdf]

S2 Appendix. Topic guide/ Interview guide

Version 1.0

Interview guide (English)

In-depth phone interview guide questions.

Thank you once again, I would like to ask you a few questions. Please feel free to let me know in case you don't wish to answer any of the questions.

1. **From the records we have on your file, you stopped coming to Mulago hospital hypertension clinic (state the date of last visit), what was the reason?**
2. **What did you know about hypertension, its treatment and control etc. before you came to Mulago hospital hypertension clinic?**
3. **When you came to the clinic what were you told about hypertension, its treatment, control and counselling etc. by the person who worked on you (Nurse, doctor or counsellor)?**

We have come to the end of the interview; I would like to thank you for your time and participation.

I have no further question. Do you have any question for me?

Respond to the questions accordingly

End call by saying" thank you once again for your participation. Have a nice day!"

MAKERERE UNIVERSITY
SCHOOL OF MEDICINE
INITIAL APPROVAL
VALID UNTIL
30 MAY 2024
RESEARCH & ETHICS COMMITTEE
P.O. BOX 7072,KAMPALA
