# Supplementary material for: Attrition and associated factors among patients on chronic antihypertensive therapy at Mulago hospital, Uganda: A mixed method study
Source: PLoS One. 2026 Feb 26;21(2):e0327933. doi: 10.1371/journal.pone.0327933 (PMC12944796; doi:10.1371/journal.pone.0327933)
Supplement: S3 Appendix — (PDF) [file pone.0327933.s003.pdf]

S3 Appendix: Graphical and statistical approaches used to assess for Cox PH assumptions

Alcohol use, **cohort year**, **drug regimen**, and **herb use** seem to violate the proportional hazards assumption because their curves cross each other, indicating they are not parallel.

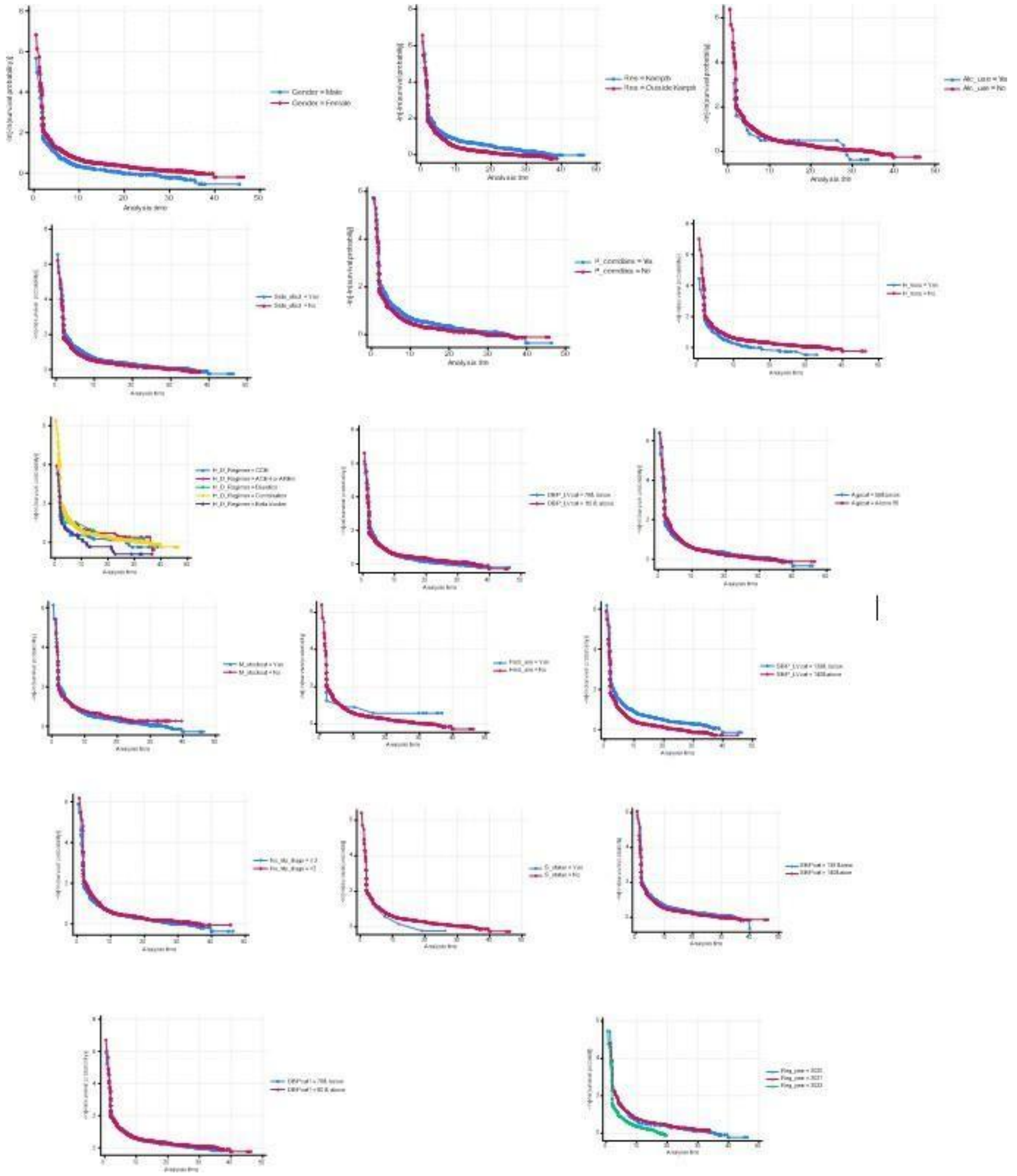

SI Fig. Log – log survival curve against time.

All variables seem to fulfill the assumption except to the small extent **herbs use and alcohol use** because the

expected and the observed do not seem to close to each other.

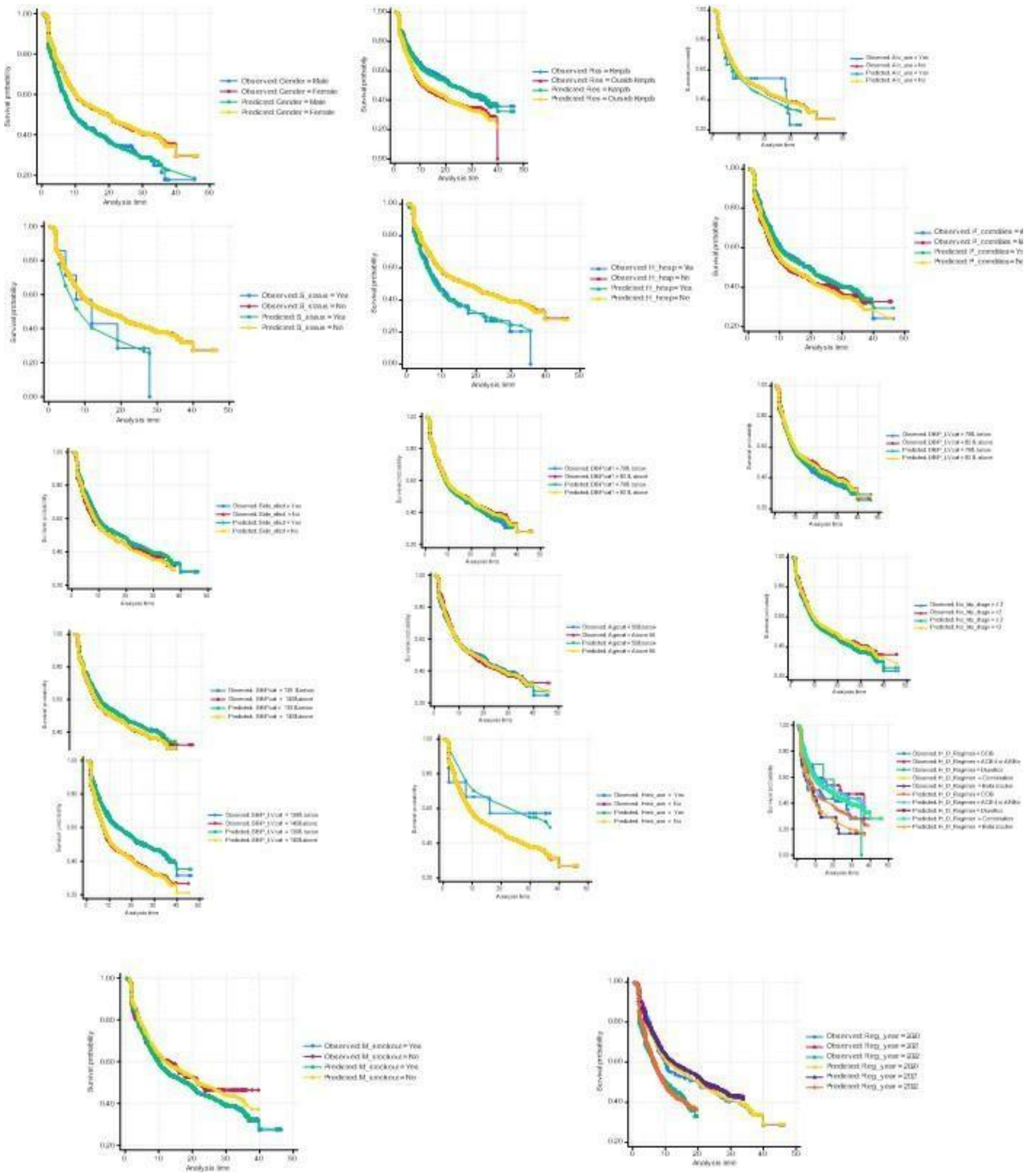

S2 Fig. Expected Vs observed survival curves

All variables seem to fulfill the assumption except to **cohort year** because the plots **are not perfectly linear**, especially at early times (left side of the plot), where they curve upward before roughly straightening out.

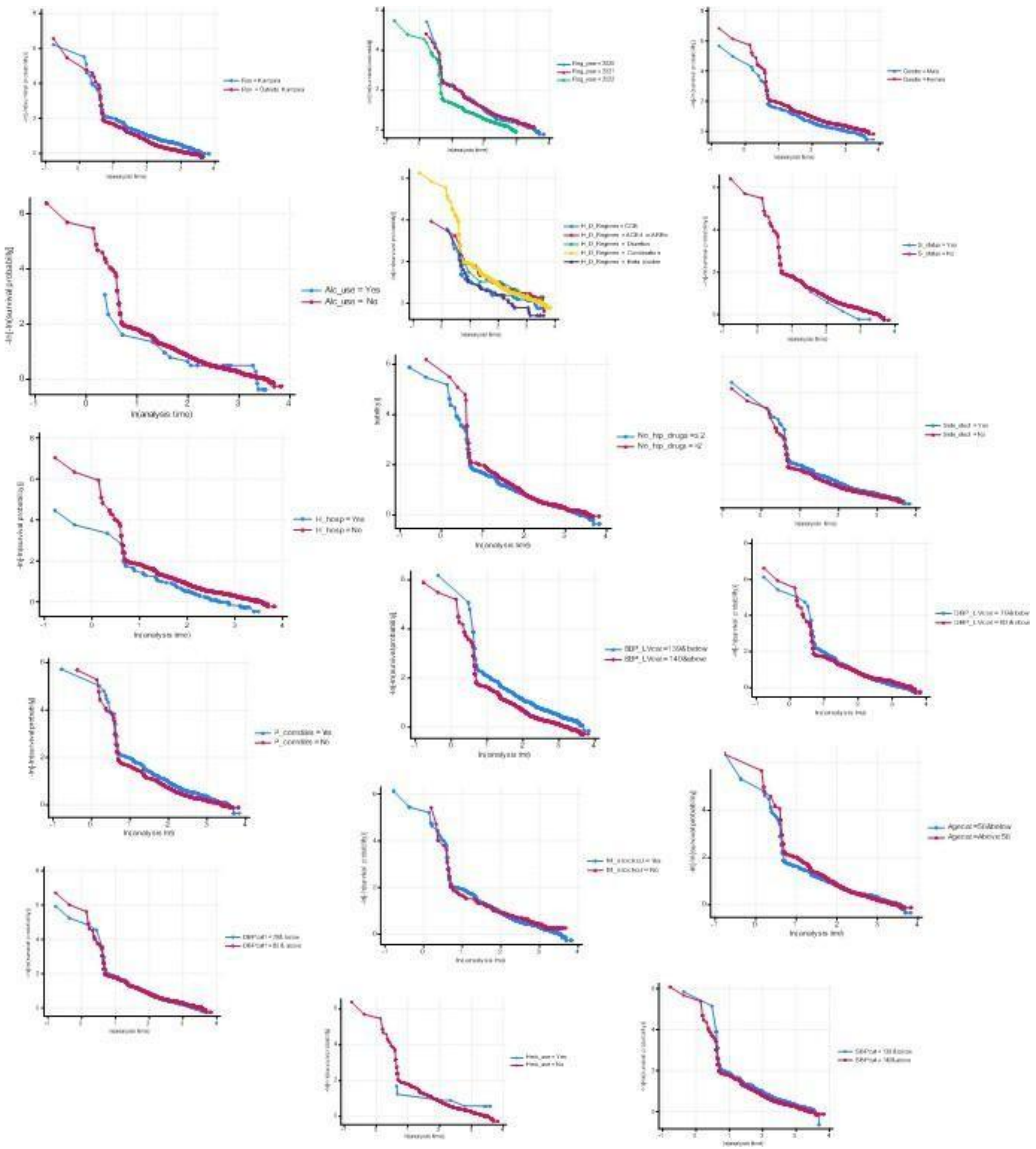

S3 Fig. log – log survival curve against log time

Since the smoothed estimate is nearly constant (horizontal) throughout, therefore all the variables seem to fulfill the PH assumption.

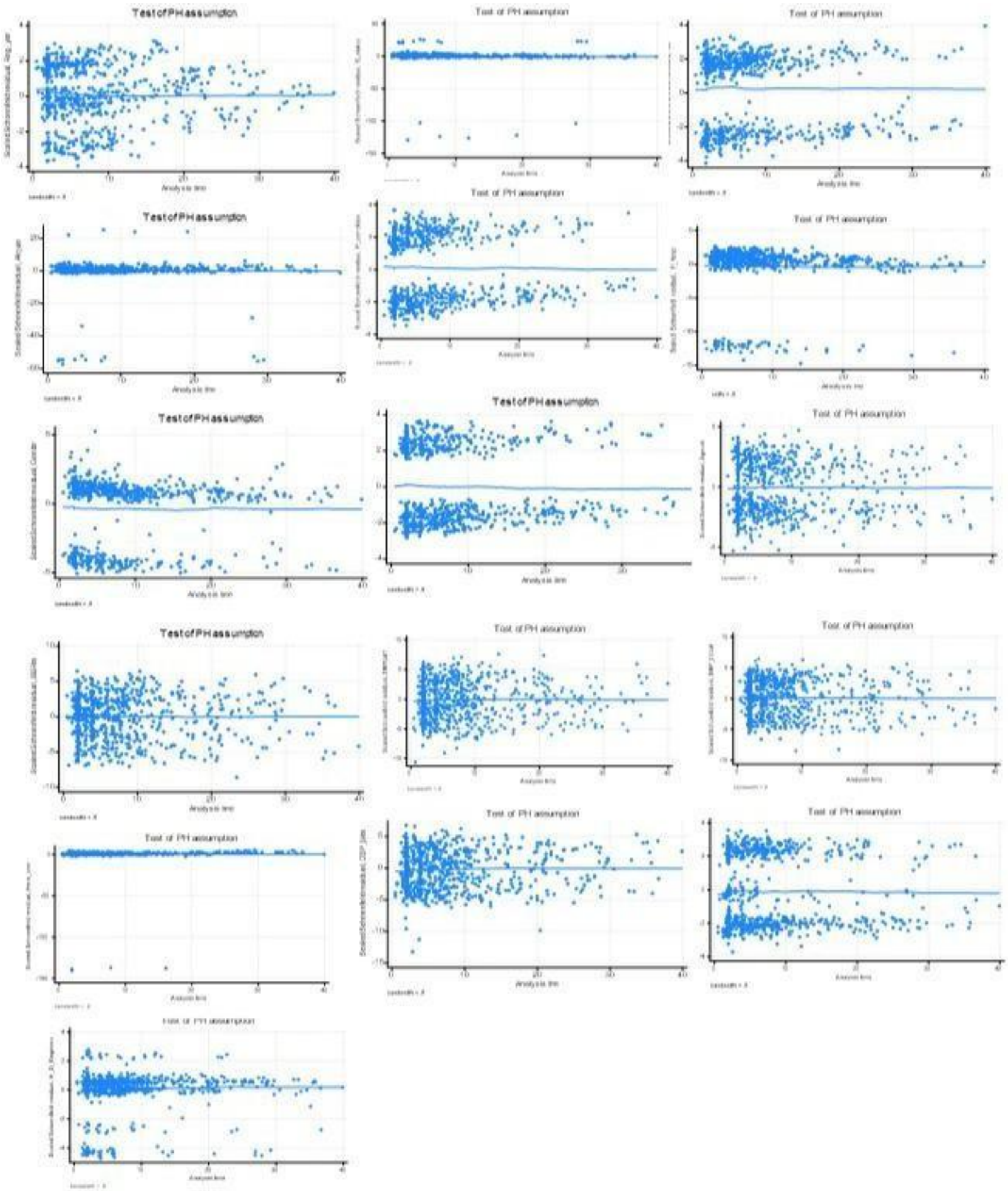

S4 Fig. Scaled schoenfeld residuals Vs Analysis time

Since the global test is statistically significant (p=0.0096) then we reject the null hypothesis that Hazard ratio is constant implying that the PH assumption not satisfied/fulfilled

Time function: Analysis time

	rho	chi2	df	Prob>chi2
Reg_year	-0.02732	0.60	1	0.4387
Res	-0.05946	2.56	1	0.1097
Gender	-0.00802	0.05	1	0.8304
S_status	-0.05762	2.39	1	0.1220
Alc_use	-0.02608	0.50	1	0.4781
SBP	-0.02949	0.50	1	0.4774
DBP	-0.00168	0.00	1	0.9669
P_comdities	-0.04721	1.59	1	0.2078
H_hosp	-0.04280	1.27	1	0.2606
Side_efect	-0.03186	0.72	1	0.3959
H_D_Regimen	0.04474	1.58	1	0.2091
No_htp_drugs	-0.03574	0.89	1	0.3463
Herb_use	0.05609	2.20	1	0.1382
SBP_LV	0.08151	3.80	1	0.0514
DBP_LV	-0.12356	10.11	1	0.0015
Global test		30.72	15	0.0096

.

S5 Fig. Schoenfeld residuals test for PH assumption
