# Supplementary material for: Attrition and associated factors among patients on chronic antihypertensive therapy at Mulago hospital, Uganda: A mixed method study
Source: PLoS One. 2026 Feb 26;21(2):e0327933. doi: 10.1371/journal.pone.0327933 (PMC12944796; doi:10.1371/journal.pone.0327933)
Supplement: S4 Appendix — (PDF) [file pone.0327933.s004.pdf]

S4 Appendix. Assessing for interaction

Since the likelihood ratio test yielded a p-value of 0.1323 ($\chi^2 = 27.11$), there was no statistically significant improvement in model fit after adding the interaction terms. Consistently, the reduced model without interaction terms was preferred based on its lower AIC and BIC values, indicating a better balance between model fit.

```
. lrtest (fullmodel) (reducedmodel), stats

Likelihood-ratio test
Assumption: reducedmodel nested within fullmodel

LR chi2(20) =  27.11
Prob > chi2 = 0.1323

Akaike's information criterion and Bayesian information criterion
```

Model	N	ll(null)	ll(model)	df	AIC	BIC
reducedmodel	1,215	-4557.307	-4479.128	16	8990.255	9071.895
fullmodel	1,215	-4557.307	-4465.575	36	9003.15	9186.84

Note: BIC uses N = number of observations. See **[R] IC note**.

S6 Fig. Chunk test
