# Supplementary material for: Attrition and associated factors among patients on chronic antihypertensive therapy at Mulago hospital, Uganda: A mixed method study
Source: PLoS One. 2026 Feb 26;21(2):e0327933. doi: 10.1371/journal.pone.0327933 (PMC12944796; doi:10.1371/journal.pone.0327933)
Supplement: S1 Table — (PDF) [file pone.0327933.s005.pdf]

S1 Table. Illustrative quote linking each theme to representative participant response

Main Theme	Sub-theme	Illustrative Quote(s)
Structural and Contextual Barriers	Preference for Alternative Sources of Medication	I'm also a health work, most of the time I get my drugs from somewhere else. My sibling at Najjanakumbi gave me some herbal medicine, that I use.
	Financial Hardship	I've been having a challenge of lack of money. I lost my job and could no longer afford to keep coming for check-ups
	Transport Barriers Exacerbated by COVID-19 Restrictions and Geographical Distance	The problem is COVID-19 came in and destabilized movement. I met some people and they told me to go to Kasese hospital for treatment because the distance to Mulago was far.
	Social Disruptions and Emotional Strain	I lost my relative and I traveled for burial and took long to come back. I don't have a caretaker, it is me who takes care of myself, my children who would take care of me are not around.
	Limited Mobility Due to Advanced Age	Being an elderly person and weak, I was tired and decided to just sit home.
	Competing Work Demands	Work is too much at the specialized hospital where I work, a few times I visited clinic people at work complained thinking I had gone to work somewhere else.
Health System Barriers	Overcrowding and Long Waiting Times	Sometimes you reach at the clinic and you are made to stay in the queue for so long. I came to the clinic, there were very many patients and we would spend a lot of time there.
	Perceived Medical Rudeness and Unfriendly Provider Attitudes	Most of the doctors are rude, you ask them a question and he is rude. Doctors are not willing to help.
	Recurrent Stockouts of Prescribed medication	Even when you get transport money, you don't find medicine
	Frustration with Appointment Scheduling and Inaccessible Care	I was told to go see some doctor but I couldn't find him for three weeks
Illness Perceptions and Health-Related Limitations	Physical Limitations Due to Hypertension-Related Complications	I stopped coming because I had a stroke and so it was hard for me to move
	Perceived lack of Treatment Effectiveness	I would not get any change after getting the medication
	Perceived Wellness and the Absence of Symptoms	If I'm feeling well, is there need to come back to the clinic. I don't feel sick.
