# Supplementary material for: Attrition and associated factors among patients on chronic antihypertensive therapy at Mulago hospital, Uganda: A mixed method study
Source: PLoS One. 2026 Feb 26;21(2):e0327933. doi: 10.1371/journal.pone.0327933 (PMC12944796; doi:10.1371/journal.pone.0327933)
Supplement: S2 Table — (PDF) [file pone.0327933.s006.pdf]

S2 Table. Joint Display Linking Quantitative Predictors to Qualitative Themes

**Quantitative predictors**	**Direction of association**	**Qualitative themes**
Age	Higher attrition (HR < 1 per year increase)	Structural and Contextual Barriers
Sex (male)	Higher attrition (HR for female < 1, protective)	Structural and Contextual Barriers
Outside Kampala residence	Higher attrition (HR > 1)	Structural and Contextual Barriers
Cohort entry year (2022)	Higher attrition (HR > 1)	Health System Barriers
Last visit SBP (Higher)	Higher attrition (HR >1mmHg increase)	Illness Perceptions and Health-Related Limitations
Last visit DBP (lower)	Protective (HR < 1mmHg increase)	Illness Perceptions and Health-Related Limitations

HR = Hazard Ratio; SBP = Systolic Blood Pressure; DBP = Diastolic Blood Pressure. Themes were derived inductively from qualitative interviews with patients lost to follow-up
