# Supplementary material for: Attrition and associated factors among patients on chronic antihypertensive therapy at Mulago hospital, Uganda: A mixed method study
Source: PLoS One. 2026 Feb 26;21(2):e0327933. doi: 10.1371/journal.pone.0327933 (PMC12944796; doi:10.1371/journal.pone.0327933)
Supplement: Attachment — Submitted filename: report_10.1101+2025.06.27.25330319.pdf [file pone.0327933.s007.pdf]

ScreenIT Report

This is an automated report for 10.1101+2025.06.27.25330319.pdf. This report was solicited by the PLOS One editorial team and provided by ScreenIT.

ScreenIT is an independent group of scientists developing automated tools that analyze academic papers. A set of automated tools screened your submitted manuscript and provided the report below. Each tool was created by your academic colleagues with the goal of helping authors.

The tools look for factors that are important for transparency, rigor and reproducibility, and we hope that the report might help you to improve reporting in your manuscript. Within the report you will find links to more information about the items that the tools check. These links include helpful papers, websites, or videos that explain why the item is important.

While our screening tools aim to improve and maintain quality standards they may, on occasion, miss nuances specific to your study type or flag something incorrectly. Each tool has limitations that are described on the ScreenIT website. The tools screen the main file for the paper; they are not able to screen supplements stored in separate files.

Please note that the Academic Editor had access to these comments while making a decision on your manuscript. The Academic Editor may ask that issues flagged in this report be addressed.

If you would like to provide feedback on the ScreenIT tool, please email the team at ScreenIt@bih-charite.de. If you have questions or concerns about the review process, please contact the PLOS One office at plosone@plos.org.

How to read the report:

- no action required

?
- review for potential issues

!
- a critical issue was found by a tool

n/a
- may not be applicable to your paper

- more information about this item can be obtained using this link

Rigor

✓	Ethics	Consent: "For the qualitative component of the study, two trained and registered clinic nurses contacted patients who had been confirmed as lost to follow-up (LTFU) and guided them through the informed consent process."<br>IRB: "Ethical approval was obtained from the Institutional Review Board (IRB) through the School of Medicine Research and Ethics Committee (SOMREC) of Makerere University College of Health Sciences (Mak-SOMREC-2023-584)."
✓	Inclusion and Exclusion Criteria	"Patients files missing data on more than 30% of the studied variables were excluded."
?	Flow charts and attrition	We did not find a study flow chart of excluded observations. We strongly recommend using flow charts because they provide an overview of the study design and more information about attrition. If you included a study flow chart in your supplemental files, we apologize for missing this. Our tool is not able to screen separate supplemental files.<br><br>Sentence about attrition: "Results Among the 1,278 patients registered at the Mulago Hypertension Clinic (20202022), 63 were excluded due to missing more than 30% of study data."
✓	Sex as a biological variable	"Characteristics of patients registered for anti-hypertensive therapy at the Mulago hospital from January 2020 to December 2022 More than half of the patients resided outside Kampala (58.8%, n= 714) and majority were female (76.1%, n= 924), with mean age of 56.5 years (SD 13.9)."
✓	Subject Demographics	Age: "Ratio of those aged 53-65 to those aged 41-52 (54/53) 20 e1= Expected events in those aged 41-52 21 e2=Expected events in those aged 53-65 22 E= Number of events 23 Substituting in the above formulae, 238 events were gotten."

?	Randomization	Not detected. If you performed an experimental study, please specify whether participants, animals or samples were randomly assigned to treatments or groups, and specify the randomization procedure.
?	Blinding	Not detected. Please specify whether blinding was used at various phases of the experiment (e.g., blinding of patients, caregivers, outcome assessments, data analysis).
✓	Power Analysis	"106 To determine the factors associated with time to attrition, the sample size was estima 107 for survival data sample size calculation."
n/a	Replication	not required.

Generated by Barzooka (flow charts), SciScore (everything else). SciScore only screens the text of the methods section; we apologize if the tool has missed items reported elsewhere in the paper or supplements.

Resources

No key resources detected.
Generated by SciScore. SciScore only screens the text of the methods section; we apologize if the tool has missed items reported elsewhere in the paper or supplements.

Transparency

?	Open code	If you wrote code to analyze your data, please consider sharing the code in a public repository. This makes it easier for others to reproduce your analyses, and may also aid others seeking to analyze similar datasets.
?	Open data	If permitted, sharing data on a public repository with a persistent identifier (a DOI or accession number) can improve reproducibility and make it easier for other scientists to expand on your work. Papers with open data are cited more often than papers without open data. Some institutions have an expert who can provide advice on data sharing.
✓	Self-acknowledged limitations	"The qualitative findings reveal a multidimensional interplay of why patients get lost to follow-up, categorized into structural and contextual barriers, health system barriers, and

		illness perceptions and healthrelated limitations. Structural and contextual issues such as financial hardship, transportation challenges especially heightened by COVID-19 restrictions and competing work demands illustrate the broader socioeconomic constraints that hinder continuity of care, consistent with findings from prior studies in developed and low-resource settings (36-38). Health system barriers, including long waiting times, perceived provider rudeness, and frequent medication stockouts, reflect systemic inefficiencies that erode patient trust and motivation, these findings are similar to what is reported in South Africa and in a narrative review conducted by systematically searching electronic databases (39, 40). Additionally, patients' perceptions of being well and symptom-free, coupled with phys..."
	Conflict of interest statement	Thank you for stating any conflicts of interest.
	Funding statement	Thank you for reporting your funding source(s), or stating that there was no specific funding for this work.
n/a	Registration statement	A registered protocol makes the planned research project more transparent and can protect against bias. Please consider registering future confirmatory studies.

Generated by ODDPub, limitation-recognizer, rtransparent, trial-identifier.

Figures

	No bar graphs of continuous data	Thank you for not using bar graphs to present continuous data. Many different datasets and data distributions can lead to the same bar graph, and the actual data may suggest different conclusions from the summary statistics alone.
	No rainbow color maps	Thank you for not using rainbow colormaps. Rainbow color maps distort readers’ perception of the data by introducing visual artifacts and are not accessible to colorblind readers.

Generated by JetFighter, Barzooka.
