# Supplementary material for: Attrition and associated factors among patients on chronic antihypertensive therapy at Mulago hospital, Uganda: A mixed method study
Source: PLoS One. 2026 Feb 26;21(2):e0327933. doi: 10.1371/journal.pone.0327933 (PMC12944796; doi:10.1371/journal.pone.0327933)
Supplement: Attachment — Submitted filename: Rebuttal_letter_NN.docx [file pone.0327933.s009.docx]

**Response to reviewer’s comments**

**Title**: Attrition and associated factors among patients on chronic antihypertensive therapy at Mulago hospital, Uganda: A mixed method study

**Corresponding Author:** Nathan Ntenkaire

**Date**: 29^th^/10/2025

| **Number** | **Comments** | **Response** | **Line/Page number/ reference** |  |
| --- | --- | --- | --- | --- |
| **Academic editor** | | | |  |
| I | Methodological Clarity and Sampling Strategy Issue: The sampling procedure and sample size calculation contain redundant explanations and inconsistent formulas (pages 5–7). The justification for using both Kish (1965) and survival data formulas is not clearly connected to study objectives. Revision Suggestion: Clarify how the final sample size (n=1215) was derived and why it exceeds the calculated requirement. Streamline the sample size section by presenting a single, coherent formula with assumptions, parameters, and effect size justification. | We thank the reviewer for this insightful comment, which helped us improve the methodological clarity of our manuscript. The section on sample size and sampling has been revised and streamlined to remove redundancy and ensure coherence between the formulas used and the study objectives.  Specifically, the Kish (1965) formula was used to estimate the sample size for the first objective “determining the attrition proportion” while the survival data formula was used for the second objective “identifying factors associated with time to attrition”. The survival-based approach was deemed more appropriate for the analytical objective involving time-to-event data.  The final required minimum sample size was 1,006 patient files after adjusting for a 10% allowance for incomplete data. However, since we employed a consecutive sampling approach, we included all 1,215 eligible patient files from January 2020 to December 2022. This approach ensured that all available data were utilized to maximize statistical power and enhance representativeness of the study population | **101-118** |  |
| 2. | Mixed-Methods Integration and Design Justification  Issue: Although the study uses a sequential explanatory mixed-methods design, the integration of quantitative and qualitative findings is not explicitly described in the Results or Discussion sections.  Revision Suggestion: Add a section explicitly describing how qualitative results complemented quantitative findings (e.g., a joint display or integrated interpretation paragraph). State how qualitative insights influenced the interpretation of statistical results. | Though we had integrated the quantitative and qualitative findings in the discussion.  We have addressed the reviewer’s concern by adding a dedicated section titled “Integration of Quantitative and Qualitative Findings” in the Results. This section explicitly describes how the qualitative findings complement and enrich the interpretation of the quantitative results.  These integrated findings provide a richer understanding of attrition drivers and demonstrate how qualitative insights can inform and contextualize quantitative results, enhancing the interpretation of statistical analyses.  We appreciate the reviewer’s insightful comment, which helped us strengthen the presentation and interpretation of our mixed-methods findings. This integrated approach enhances the rigor and depth of our study, providing a clearer justification for the sequential explanatory mixed-methods design employed. | **Line 333-344** |  |
| 3. | Statistical Rigor and Model Selection  Issue: The rationale for using an Extended Cox Regression Model is mentioned (violation of proportional hazard assumption) but not shown graphically or statistically. Furthermore, the proportional hazards test results are not reported.  Revision Suggestion: Include diagnostic plots or a supplementary figure/table demonstrating the proportional hazard assumption violation. Provide the name of the variable that violated the assumption and justify the model extension quantitatively | Thank you for this constructive comment. We have now provided both graphical and statistical evidence to demonstrate the violation of the proportional hazards (PH) assumption and have justified our use of an extended Cox regression model.  Specifically:  We assessed the PH assumption graphically. Variables like alcohol use, cohort entry year, herbs use, drug regimen seemed to violate the Cox PH assumption. The corresponding diagnostic plots are now included in the Supplementary Information (S2 Appendix).  A Schoenfeld residuals test was conducted. The test indicated that last visit DBP violated the PH assumption (p < 0.0015). With an overall global test (chi^2^_=30.76, P= 0.0096_). indicating violation of the Cox PH model as a whole, hence The extended model demonstrated better fit.  Because of these violations, we extended the Cox model to allow time-varying effects for these variables, however only the last-visit DBP time varying covariate remained significant.  We have added a detailed explanation in the Method and referenced the supplementary diagnostic plots and test statistics accordingly. | **165-194**  **S2 Appendix** |  |
| 4. | Validity and Reliability of Qualitative Analysis  Issue: The qualitative phase describes thematic analysis with independent coders, but there is no mention of intercoder reliability or saturation confirmation process beyond “data saturation was achieved after 16 interviews.”  Revision Suggestion: Specify whether intercoder agreement was measured (e.g., Cohen’s kappa) and describe the analytical validation process (peer debriefing, triangulation, or audit trail). Add an illustrative quote table linking each theme to representative participant responses. | Thank you for this insightful comment. We appreciate the suggestion to clarify how validity and reliability were ensured in the qualitative phase. We have now expanded the Data Analysis subsection to describe the measures taken to ensure analytical rigor. Specifically, we have indicated that two independent coders conducted the thematic analysis and resolved any coding discrepancies through consensus discussions, rather than statistical measures such as Cohen’s kappa. We have also elaborated on the validation strategies used, including peer debriefing throughout the analysis process. Additionally, we have clarified how data saturation was determined and have developed an illustrative table linking each major theme with representative participant quotes (see S1 Table). | **187-194**  **S1 Table”** |  |
| 5 | Over interpretation of Quantitative Findings  Issue: The conclusion implies that factors such as age and male sex cause attrition, which overstates the observational design. Phrases like “patients who resided outside Kampala were 31% more likely to get lost to follow-up” may imply causality rather than association.  Revision Suggestion: Revise language to emphasize associations and avoid causal implications. For example, use “was associated with” instead of “led to” or “caused.” Include a clear statement acknowledging the limitations of observational inference. | Thank you for this valuable observation. We have revised the results, discussion, and conclusion sections to avoid causal language and to emphasize that findings represent associations rather than causal effects. We have also added a statement in the limitations section acknowledging that the observational design precludes causal inference. | **Discussion**  **Study strength & limitation “section** |  |
| 6 | Contextualization with Existing Literature  Issue: The discussion references multiple unrelated chronic disease studies (e.g., HIV, diabetes) without sufficient justification for their analogy to hypertension care. Regional literature from sub-Saharan Africa on hypertension retention is underrepresented.  Revision Suggestion: Strengthen discussion by citing and comparing results with regionally relevant hypertension adherence studies (e.g., Nigeria, Kenya, Tanzania). Discuss context-specific barriers such as drug stockouts or urban–rural disparities using LMIC evidence. | Thank you for this valuable comment. We agree that contextualization with regionally relevant literature is important. In revising the discussion, we clarified that, to the best of our knowledge, no studies in sub-Saharan Africa have specifically reported on attrition from hypertension care. Most available studies from the region focus on medication adherence among hypertensive patients, which, although related, is conceptually distinct from attrition.  Despite the absence of directly comparable attrition studies, we have strengthened the discussion and we have also minimized reliance on analogies from unrelated chronic disease literature |  |  |
| 7 | Writing Structure and Presentation  Issue: The manuscript is lengthy and contains redundant phrases (e.g., repeated mention of “Mulago hospital” and “chronic antihypertensive therapy”). Tables are numerous but lack clarity on the meaning of abbreviations, and figure legends (e.g., Fig 2) are insufficiently described.  Revision Suggestion: Streamline narrative flow by merging repetitive sentences and ensuring all abbreviations are defined upon first use. Improve figure/table captions for stand-alone comprehension. Move extensive methodological formulas and supplementary data into appendices. | We thank the reviewer for the feedback. We have streamlined the manuscript by removing redundant phrases, defined all abbreviations at first use, and revised figure and table legends for stand-alone clarity. Extensive methodological formulas and supplementary data have been moved to the appendices to improve readability and flow. |  |  |
| **Reviewer two** | | | |  |
| 1 | Outcome definition & time origin. Attrition is defined as ≥2 consecutive missed appointments. Please specify the usual appointment interval (e.g., monthly/bi-monthly) and the exact time origin for survival analyses (e.g., clinic registration or first antihypertensive prescription) so readers can understand person-time construction and the practical meaning of “two consecutive” misses. | Thank you for this helpful comment. We have clarified that follow-up appointments were not uniform, as each patient was given individualized schedules based on their clinical condition and treatment response. We also specified that the time origin for survival analysis was the date of registration into chronic hypertensive care, from which person-time was computed until attrition or end of follow-up (date of data collection) | **Check variables section** |  |
| 2 | Survival modelling details. You report Kaplan–Meier and Cox (with proportional hazards checks and use of an extended Cox model when needed). Please: (a) state which variables violated PH and how you addressed this; (b) provide PH diagnostics (tests/plots) in the supplement; (c) describe model specification clearly candidate variables, criteria for entry/retention, handling of interactions and confounding. | Thank you for this helpful comment. We have revised the Data analysis section to include more detail on model specification and diagnostic procedures. Specifically, we clarified that the proportional hazards assumption was assessed both graphically and statistically using Schoenfeld residual test. The tests indicated that the assumption was violated for the model overall (p = 0.0096) and for last-visit diastolic blood pressure (DBP) (p = 0.0015). This variable was therefore modeled as a time-varying covariate in an extended Cox model.  We also described the criteria for variable inclusion variables with p ≤ 0.2 at bivariate analysis and those known from literature were considered for the multivariable model. Interaction and confounding were assessed using chunk tests or likelihood ratio test (p=0.1323 (χ² = 27.11) (**S3 Appendix**) and a 10% change-in-estimate criterion, respectively. The PH diagnostic plots and statistical outputs have been included as **S2 Appendix*.*** | **Data analysis section,**  **S3 Appendix**  **S2 Appendix** | |
| 3 | Temporal alignment of blood pressure covariates. SBP/DBP measured at “last visit” are used as predictors. If serial measures exist, consider modelling SBP and DBP as time-varying covariates to avoid bias from values recorded close to the attrition event. If only fixed values are used, justify this choice and discuss limitations; a sensitivity analysis using baseline or penultimate-visit values would be helpful. | Thank you for this insightful comment. In this study, both baseline and last-visit blood pressure measurements were included to represent patients’ initial and most recent clinical status. The proportional hazards assumption was assessed for these variables; last-visit diastolic blood pressure (DBP) violated the assumption and was therefore modeled as a **time-varying covariate** in the extended Cox regression model. Baseline SBP and DBP, and last-visit SBP (which met the PH assumption), were modeled as **fixed covariates**. This clarification has been added to the Data analysis section, and a note acknowledging potential residual bias due to timing of last-visit measurements has been included in the Discussion*.* | **Data analysis**  **Discussion section** |  |
| 4 | Scaling and interpretation of continuous predictors. Report the units for continuous covariates in the model (e.g., age per 1 or 10 years; SBP/DBP per 1 or 10 mmHg) and ensure the text, tables, and conclusions reflect the chosen scaling. | Thank you for this helpful observation. Continuous variables were modeled per 1-unit increase specifically, age per year and systolic/diastolic blood pressure (SBP/DBP) per mmHg. This has been clarified in the Data analysis section and noted in the footnote of **Table 4**. The Results and Discussion sections were reviewed to ensure consistency in interpretation with this scaling. | **Discussion and results** |  |
| 5 | Rate” vs “proportion.” Where you present the overall percentage disengaged, please refer to it as an attrition proportion/percentage. Reserve “rate” for person-time metrics (events per person-months/years) if you also estimate those. | Thank you for this clarification. All instances of “attrition rate” have been revised to “attrition proportion” throughout the manuscript, including the Abstract, Results, and Discussion sections. The term “rate” is now reserved exclusively for person-time measures, in line with reporting standards. |  |  |
| 6 | Missing data. Beyond excluding records with high overall missingness, please describe variable-level missingness and your approach (complete-case, missing-indicator, or multiple imputation). If feasible, add a sensitivity analysis (e.g., multiple imputation) for key predictors. | Thank you for this helpful comment. Variable-level missingness was minimal. Specifically, diastolic blood pressure (DBP) at last visit was missing for 5 participants; these values were imputed using the median DBP value (83 mmHg), and age was missing for 6 participants but was computed from their recorded dates of birth. Overall, missingness across variables was less than 1%. Analyses were therefore conducted using a **complete-case approach** after median imputation for DBP. Given the negligible level of missingness, multiple imputation and sensitivity analyses were not deemed necessary. These details have been added to the Data management |  |  |
| 7 | Presentation of survival results. Add numbers-at-risk beneath Kaplan–Meier curves (or report in figure legends/tables), include log-rank p-values where curves are compared, and report median follow-up and total events to contextualize information content. | We thank the reviewer for the suggestion. The requested updates have now been incorporated: **numbers-at-risk are shown beneath the Kaplan–Meier curves, log-rank p-values are included where curves are compared, and median follow-up time along with total events are reported** in the Results section | **Result section**  **Fig 1** |  |
| 8 | Qualitative reporting & integration. Methods are concisely described; to align with COREQ expectations, please expand on reflexivity (interviewer roles/position), sampling and recruitment, language/translation, and how thematic saturation was judged. A joint display linking quantitative predictors (e.g., distance/residence) to qualitative themes (transport costs, stock-outs, clinic processes) would strengthen integration and the discussion of mechanisms. | Thank you for this constructive comment. We agree that additional detail was needed to align our qualitative methods with COREQ standards and to strengthen integration of the quantitative and qualitative components. In response, we have expanded the Methods section to provide clearer descriptions of (1) reflexivity, including interviewer roles and positionality; (2) sampling and recruitment procedures; (3) the language and translation process; and (4) how thematic saturation was determined.  Additionally, we strengthened integration of findings by adding a joint explanatory narrative that links quantitative predictors (e.g., residence outside Kampala, sex, Cohort entry year and last-visit BP measures) with qualitative themes (e.g., transport barriers, stock-outs, clinic processes, and perceptions of wellness). We also added a joint display **(S2 Table)** summarizing how qualitative themes help to explain the mechanisms underlying the quantitative associations. |  |  |
| 9 | Calendar time and external shocks. Because disengagement may vary by year (e.g., service disruptions), consider modelling calendar period (or include it as a covariate) and/or discuss how temporal shocks could influence retention. | We thank the reviewer for this insightful comment. **Cohort entry year has been included as a covariate in our models**, addressing the potential influence of calendar period and temporal shocks on retention. |  |  |
| 10 | Contextual claims and citations. Where you compare observed retention/attrition to programmatic “targets,” please provide a supporting citation and clarify whether such targets apply specifically to hypertension care. | Thank you for this comment. We have now clarified the basis for the programmatic target used in our comparisons and cited them accordingly |  |  |
| 11 | Minor comments (clarity, style, formatting) • Ensure consistent terminology (e.g., “loss to follow-up” with hyphens; consistent use of BP/SBP/DBP). • Standardize units and spacing (e.g., “mmHg”, “%”). • In the setting description, use “intensive care” rather than “intensive” alone. • Define all abbreviations at first use in the abstract and main text. • Tables: label units and scaling in headers (e.g., “HR per 10 years of age,” “HR per 10 mmHg SBP”). • Consider adding a simple flow diagram (records screened → eligible → included → retained vs LTFU/other outcomes). | We thank the reviewer for these helpful comments. All requested edits have been made. Terminology has been standardized (e.g., consistent use of “loss-to-follow-up,” BP/SBP/DBP), and units and spacing have been harmonized (e.g., “mmHg,” “%”). Abbreviations are now defined at first use, table headers indicate units and scaling, and the setting description has been revised to “intensive care.” A flow diagram showing records screened, eligible, included, and outcomes (retained vs. LTFU) is already included as study profile (Fig 1) | Fig 1 |  |
| 12 | Data, code, and reproducibility  The anonymized dataset provided in the Supporting Information is a strong step toward compliance with the PLOS Data policy. For maximal reproducibility, please also share the analysis code (e.g., do-files/scripts) and a brief data dictionary, ideally in a public repository with a stable link/DOI referenced in the Data Availability Statement. | We thank the reviewer for this valuable suggestion. In line with the PLOS Data policy, the anonymized dataset, analysis code, and data dictionary have been deposited in Zenodo, and a stable DOI link has been added to the Data Availability Statement  (<https://doi.org/10.5281/zenodo.17553717>). |  |  |
| 13 | Ethics Ethical approvals and consent/waiver procedures are described. Please ensure the ethics section clearly distinguishes approvals for the retrospective records review and for the qualitative interviews (including consent procedures, language, and confidentiality safeguards). | We thank the reviewer for this comment. We have revised the Ethics section to clearly distinguish the approvals for the two components of the study. Specifically, we now describe separate approvals for the retrospective records review and the qualitative interviews. For the records review, confidentiality and anonymity procedures are detailed, and for the qualitative interviews, we describe the consent process, the language used for study information, and measures taken to ensure confidentiality, including anonymized transcripts and secure data storage. |  |  |
| **Reviewer three** | | | |  |
| 1 | Please provide the definition you used of Lost to follow up. | Thank you for the comment. We have now clarified this in the Abstract section by defining both attrition and loss to follow-up. Specifically, attrition was defined as patients who were lost to follow-up, and loss to follow-up was defined as patients who missed two or more consecutive clinic appointments. | **Abstract section** |  |
| 2 | Please provide an overview of variables extracted from patient files | Thank you for this comment. The overview of variables extracted from patient files is provided in the Variables section of the manuscript. Specifically, we detailed the dependent and independent variables abstracted from patient records. The dependent variable was time to attrition, while the independent variables included:   - **Patient-related factors:** age, sex, residence, occupation, alcohol use, smoking status, herbal medicine use, and marital status. - **Clinical-related factors:** baseline systolic and diastolic blood pressure, presence of comorbidities, history of hospitalization, number and type of antihypertensive drugs, drug-related side effects, and last-visit blood pressure measurements. - **Health system-related factor:** medicine stock-out. | See the variable section |  |
| 3 | Please provide the topic guide and explain what topics were discussed during the interviews and if there was any theory the questions were based on. | Thank you for this comment. The topic guide (interview guide) used in the qualitative phase has now been provided as supplementary material (S7 Appendix). It included open-ended questions exploring patients reasons for loss to follow up. The questions were not based on any specific theoretical framework and therefore themes emerged from the data collected. | **See S7 Appendix** |  |
| 4 | Please ensure LTFU is abbreviated the first time it's used and the abbreviation is used consistently | Thank you for this helpful observation. We have revised the manuscript to ensure that loss to follow-up (LTFU) is written in full the first time it appears and that the abbreviation LTFU is used consistently throughout the text |  |  |
| 5 | Please provide information about recruitment of participants for interviews: how were they identified, when and where were they asked to participate, what was sampling based on? | Thank you for this comment. Details regarding participant recruitment for the qualitative phase have been clarified in the Methods section (sample size and sampling procedure, study design and population and data collection). Briefly, participants were identified from clinic records as patients confirmed to be lost to follow-up. Recruitment occurred between **20th and 28th January 2024**, when trained clinic nurses contacted eligible patients by phone and invited them to participate. Sampling was purposive, with maximum variation based on age, sex, residence, presence of comorbidities, and adverse drug effects. Recruitment continued until thematic saturation was reached. |  |  |
| 6 | In terms of the discussion, the literature comparisons are not the clearest. There are some very long sentences with multiple studies addressed (see 344-350). Additionally, some of the explanations about why data you collected differed from literature data are insufficient, for example: | Thank you for this helpful comment. We have revised the section to improve clarity and readability. In the updated version, we separated the long sentence into shorter, more focused statements and clearly presented each comparative study. |  |  |
| 7 | Line 370: do you have a reference for this? Unsure what this is based on. | We have now added the appropriate reference to support the statement that women are more likely to seek medical attention than men. |  |  |
| 8 | Line 377 to 380: I am unsure what is meant here. Most patients being adult and elderly does not seem to be an explanation for age as a reason for loss to follow up. Please clarify. | We have made it clear that Elderly patients tend to be concerned about their health compared to young individuals who exhibit less interest as an explanation for age as a reason for loss to follow up. However, the statement” most patients being adult and elderly” was an explanation for the disparity in the qualitative and quantitative findings |  |  |
| 9 | Line 381 to 387: This is unclear. Do you mean that time to attrition increases when systolic blood pressure increases, but decreases when diastolic blood pressure increases? That seems an interesting finding, why could that be? This is not discussed here, and the reasons provided don't seem very general, not specific for blood pressure measurements. | Thank you for this helpful comment. We agree that the original wording was unclear and did not adequately explain the differing associations of systolic and diastolic blood pressure with attrition. We have revised the text to clearly distinguish the effects of systolic and diastolic blood pressure and to provide a more specific and clinically grounded interpretation. The revised paragraph now explains that higher systolic blood pressure may prompt closer clinical monitoring or greater perceived risk, which could delay attrition, whereas elevated diastolic blood pressure may be perceived as less severe, leading to earlier disengagement. |  |  |
| 10 | Line 388 to 392: You first mention PLWHIV is a similar population to your population, before explaining the difference in outcomes between your study and the study in China being due to the latter having a different population. Please rectify and provide additional reasons for the difference | Thank you for highlighting this inconsistency. We have revised the paragraph to clarify that although hypertension and HIV are both chronic conditions requiring long-term medication adherence, the patient populations differ in several important ways. These differences include treatment complexity, perceived disease severity, stigma, and the frequency and structure of follow-up care. Such contextual variations may explain why drug regimen was associated with attrition in the HIV study but not in our hypertensive cohort |  |  |
| 11 | Various discrepancies in the discussion section are explained due to the referenced studies having different populations. These studies may thus not be the best to compare your findings with and would be good to see if there are better suited studies out there, or maybe the data is not comparable as such and the paragraphs should be rewritten to reflect this. | Thank you for this helpful comment. We agree that comparing our findings with studies involving substantially different populations may limit interpretability. Upon re-examining the literature, we did not identify hypertension studies directly comparable to our patient population or study outcomes. We have therefore revised the discussion to avoid inappropriate cross-population comparisons.  In all sections where comparable hypertension evidence was lacking, we rewrote the paragraphs to clearly acknowledge this limitation and to ensure that contextual references from other chronic conditions are presented cautiously and not as direct comparators |  |  |
| 12 | Additionally the discussion is very long, particularly the comparisons with other scientific literature is taking up a lot of space.  It would be good to reflect on some of your study strengths. | Thank you for this valuable feedback. We have shortened the Discussion section by condensing comparisons with previous studies and focusing on the most relevant and recent literature. We have also added a concise paragraph highlighting the strengths of our study(Study strength and limitation), including the use of a mixed-methods design that enabled triangulation of findings with deeper insights |  |  |
| 13 | Conclusion - the impact of COVID-19 is barely discussed in the discussion but mentioned in conclusion. Would be good to reflect on the impact of the pandemic in more detail in the discussion. | We thank the reviewer for this observation. We have now expanded the discussion to provide more detail on the impact of the COVID-19 pandemic on patient attrition. Specifically, we highlight that the peak in attrition among the 2020 cohort coincided with the onset of COVID-19 in Uganda and the implementation of strict public health measures, including lockdowns, curfews, and restrictions on public transport. These measures, along with fear of exposure to COVID-19 in healthcare facilities and temporary reorganization of healthcare services, likely limited patients’ access to routine care and contributed to higher loss to follow-up |  |  |
